# Fishborne Zoonotic Trematodes Transmitted by *Melanoides tuberculata* Snails, Peru

**DOI:** 10.3201/eid2403.172056

**Published:** 2018-03

**Authors:** Eduardo A. Pulido-Murillo, Luis Fernando V. Furtado, Alan L. Melo, Élida M.L. Rabelo, Hudson A. Pinto

**Affiliations:** Universidade Federal de Minas Gerais, Belo Horizonte, Brazil

**Keywords:** trematodes, Heterophyidae, zoonoses, Melanoides tuberculata, fishborne disease, Peru, snails, parasites

## Abstract

We investigated the transmission of the fishborne trematodes *Centrocestus formosanus* and *Haplorchis pumilio* by *Melanoides tuberculata* snails in Peru. We report on results of experimental, morphological, and molecular approaches and discuss the potential risk for future human cases, given the existence of food habits in the country involving the ingestion of raw fish.

The World Health Organization has estimated that the number of humans infected with fishborne trematodes exceeds 18 million, and >500 million persons are at risk of infection (*1*). Among the causative agents of these trematodiases are representatives of the family Heterophyidae, which are small intestinal parasites from birds and mammals, including humans (*1**–**3*). Infection by heterophyids can be considered an emerging disease because of a set of factors, including high prevalence, reported mainly in Asia; outbreaks caused by *Ascocotyle longa* trematodes in Brazil; the introduction of *Centrocestus formosanus* trematodes and *Haplorchis pumilio* flukes from Asia into the Americas; and the involvement of larvae of *Procerovum varium* flukes as causative agents of human ocular disease (*3**–**5*).

Of the 30 species of heterophyids recognized worldwide (*4*), 7, including *C. formosanus* and *H. pumilio*, are transmitted by the red-rimmed melania or Malaysian trumpet snail, *Melanoides tuberculata* (*6*). Even though human infection by these 2 heterophyids has not been reported in the Americas, the possibility of future cases must be considered, especially in countries like Peru, whose inhabitants consume ceviche, a culinary dish prepared with raw fish. Thus, the evaluation of the involvement of *M. tuberculata* snails in the transmission of heterophyids in this country is needed given the potential public health concern related to these parasites.

In this study, we collected snails in 2 areas of the central coast of Peru, the Ventanilla Wetlands Regional Conservation Area (VWRCA) (11°52′31″S; 77°8′37″W) and the Pantanos de Villa Wildlife Refuge (PVWR) (12°12′33″S; 76°59′28″W), during December 2015 and January, June, and July 2016. Identification of the collected *M. tuberculata* snail specimens was based on conchiliological features according to previously published reports (*7**,**8*). We placed the specimens individually onto polystyrene plates containing dechlorinated water, subjected them to photostimulation, and examined them under a stereomicroscope. We examined cercariae, fluke larvae, in a light microscope after vital staining (0.05% Nile blue sulfate) and preliminarily identified 2 cercarial types, pleurolophocercous (Figure, panel A) and parapleurolophocercous (Figure, panel E). We used samples of the cercariae for experimental infection of vertebrate hosts to obtain other developmental stages for identification. Experiments were conducted in accordance with the local animal experimentation ethics committee (Comissão de Ética no Uso de Animais, Universidade Federal de Minas Gerais, protocol 20/2016). 

**Figure:**
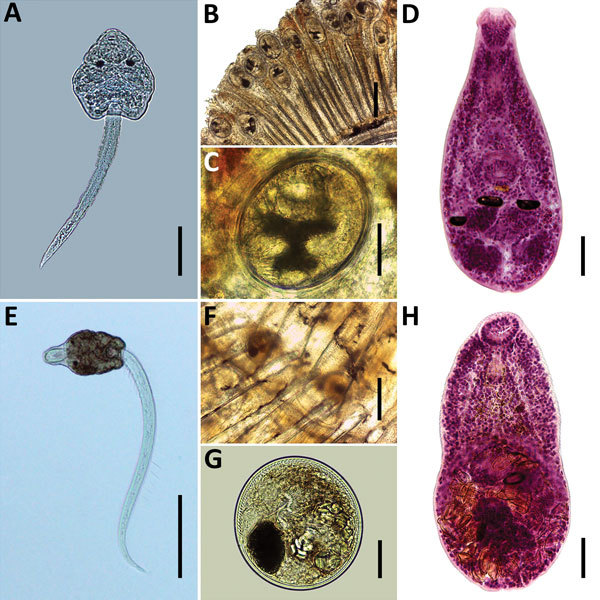
Species of heterophyids transmitted by *Melanoides tuberculata* snails in Peru. A–D) *Centrocestus formosanus*: cercaria (pleurolophocercous type) (A), encysted metacercariae in gills of *Poecilia reticulata* (B, C), and adult parasite obtained in experimentally infected mouse (D). E–H) *Haplorchis pumilio*: cercaria (parapleurolophocercous type) (E), metacercariae found at the base of the caudal fin of *P. reticulata* (F–G), and adult recovered in experimentally infected mouse (H). Scale bars indicate 50 μm in panels A, C, D, G, and H, 200 μm in panels B, E, and F.

We exposed 2 groups of *Poecilia reticulata* guppies (n = 30) individually to 50–100 cercariae of each larval type. We euthanized fish surviving at 30 days postinfection and collected metacercariae found in the gills (Figure, panels B, C) of fish infected with pleurolophocercous cercariae and in the bases of the fins (Figure, panels F and G) of fish exposed to parapleurolophocercous cercariae. We administered metacercariae orally to dexamethasone-immunosuppressed mice. Adult parasites recovered in the small intestines of mice at 6–7 days postinfection were fixed, stained, and mounted on permanent slides. We studied the morphology of the experimentally obtained stages using a light microscope for identification according to taxonomic works (*2**,**9*).

We used ethanol-fixed aliquots of cercarial types obtained in *M. tuberculata* snails for molecular characterization. We extracted DNA using the Wizard Genomic DNA Purification Kit (Promega, Madison, WI, USA) and amplified a fragment of the 28S rDNA by PCR using the primers Dig12 (forward) and 1500R (reverse) with PCR conditions as previously described (*10*). We purified the PCR products with 20% polyethylene glycol 8000 (Promega) and sequenced them in an ABI3730 automated sequencer using Pop-7 Polymer and the ABI BigDye v3.1 Cycle Sequencing Kit (Applied Biosystems, Foster City, CA, USA). We edited the sequences we obtained using ChromasPro version 2.0.1 (Technelysium Pty Ltd, South Brisbane, Queensland, Australia), compared them with data available in GenBank, and used them for phylogenetic analyses based on the maximum likelihood method using MEGA7 (http://www.megasoftware.net/) and Bayesian inference method using MrBayes 3.1.2 (http://mrbayes.sourceforge.net/). We deposited the obtained sequences in GenBank (accession nos. MG738251 and MG738252).

From the experimental infection of mice, we obtained adult parasites identified as *C. formosanus* (Figure, panel D) and *H. pumilio* (Figure, panel H). Molecular data revealed that the samples of *C. formosanus* and *H. pumilio* cercariae found in *M. tuberculata* snails from Peru are conspecific with isolates of these species from Vietnam and Thailand (99.8%–100% similarity), a finding supported by phylogenetic analyses (Technical Appendix). In total, we collected 6,731 *M. tuberculata* snails, of which 112 (1.66%) were found to be infected with heterophyid cercariae. We found *C. formosanus* cercariae in 71 (1.8%) of 3,874 snails collected in VWRCA and in 29 (1.0%) of 2,857 snails collected in PVWR. We found *H. pumilio* cercariae in 12 of 2,857 (0.4%) snails from PVWR.

The presence of *C. formosanus* and *H. pumilio* trematodes in *M. tuberculata* snails from Peru reveals the need to focus attention on the possible effect of these fishborne agents on human health. The increasing number of reports of these trematodes in the Americas indicates that the geographical areas they have invaded are expanding. This finding should serve as a warning, particularly given the popularity of dishes based on raw fish.

Technical Appendix Phylogenetic relationship between *Centrocestus formosanus* and *Haplorchis pumilio* found in Peru (shown in bold) with other heterophyids transmitted by *Melanoides tuberculata*, inferred from partial sequences of 28S rDNA (1176 bp) based on Bayesian inference (BI) and maximum likelihood (ML) analyses. For the ML analysis, nodal support was estimated by the bootstrap method (1000 replications). BI analysis was based on 2 runs, each of 4 chains based on Markov chain Monte Carlo (MCMC) analysis for 1 million generations with sampling interval of 100 generations and a burn-in of 25%. The numbers on the nodes are the posterior probability values and bootstrap for BI and ML, respectively. *Echinostoma revolutum* was used as an external group. The scale bar indicates the number of substitutions per site.
