# Supplementary material for: Fishborne Zoonotic Trematodes Transmitted by Melanoides tuberculata Snails, Peru
Source: Emerg Infect Dis. 2018 Mar;24(3):606–8. doi: 10.3201/eid2403.172056 (PMC5823359; doi:10.3201/eid2403.172056)
Supplement: Technical Appendix — Phylogenetic relationship between Centrocestus formosanus and Haplorchis pumilio found in Peru (shown in bold) with other heterophyids transmitted by Melanoides tuberculata, inferred from partial sequences of 28S rDNA (1176 bp) based on Bayesian inference (BI) and maximum likelihood (ML) analyses. For the ML analysis, nodal support was estimated by the bootstrap method (1000 replications). BI analysis was based on 2 runs, each of 4 chains based on Markov chain Monte Carlo (MCMC) analysis for 1 million generations with sampling interval of 100 generations and a burn-in of 25%. The numbers on the nodes are the posterior probability values and bootstrap for BI and ML, respectively. Echinostoma revolutum was used as an external group. The scale bar indicates the number of substitutions per site. [file 17-2056-Techapp-s1.pdf]

Article DOI: https://doi.org/10.3201/eid2403.172056

Fishborne Zoonotic Trematodes Transmitted by *Melanoides tuberculata* Snails, Peru

Technical Appendix

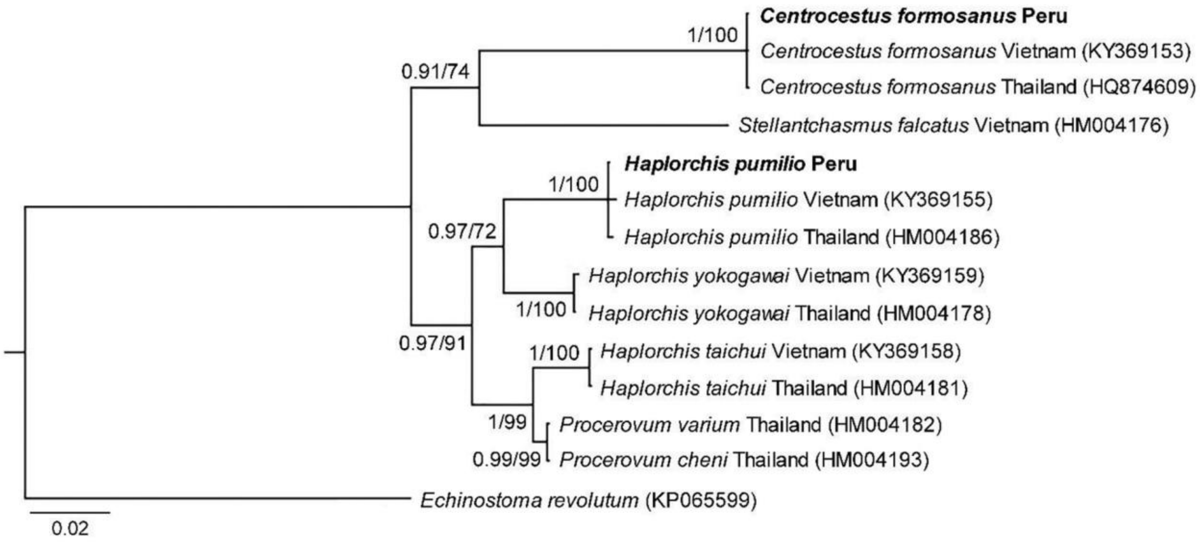

Technical Appendix Figure. Phylogenetic relationship between *Centrocestus formosanus* and *Haplorchis pumilio* found in Peru (shown in bold) with other heterophyids transmitted by *Melanoides tuberculata*, inferred from partial sequences of 28S rDNA (1176 bp) based on Bayesian inference (BI) and maximum likelihood (ML) analyses. For the ML analysis, nodal support was estimated by the bootstrap method (1000 replications). BI analysis was based on 2 runs, each of 4 chains based on Markov chain Monte Carlo (MCMC) analysis for 1 million generations with sampling interval of 100 generations and a burn-in of 25%. The numbers on the nodes are the posterior probability values and bootstrap for BI and ML, respectively. *Echinostoma revolutum* was used as an external group. The scale bar indicates the number of substitutions per site.
